# Supplementary material for: Functional connectivity subtypes associate robustly with ASD diagnosis
Source: eLife. 2022 Nov 29;11:e56257. doi: 10.7554/eLife.56257 (PMC9708070; doi:10.7554/eLife.56257)
Supplement: Transparent reporting form [file elife-56257-transrepform1.docx]

***eLife’s* transparent reporting form**

We encourage authors to provide detailed information *within their submission* to facilitate the interpretation and replication of experiments. Authors can upload supporting documentation to indicate the use of appropriate reporting guidelines for health-related research (see [EQUATOR Network](about:blank)), life science research (see the [BioSharing Information Resource](about:blank)), or the [ARRIVE guidelines](about:blank) for reporting work involving animal research. Where applicable, authors should refer to any relevant reporting standards documents in this form.

If you have any questions, please consult our Journal Policies and/or contact us: [editorial@elifesciences.org](about:blank).

**Sample-size estimation**

- You should state whether an appropriate sample size was computed when the study was being designed
- You should state the statistical method of sample size computation and any required assumptions
- If no explicit power analysis was used, you should describe how you decided what sample (replicate) size (number) to use

Please outline where this information can be found within the submission (e.g., sections or figure legends), or explain why this information doesn’t apply to your submission:

This manuscript uses data from the publicly available ABIDE 1 and 2 datasets ([http://fcon_1000.projects.nitrc.org/indi/abide/](about:blank)) and the longitudinal HNU1 dataset ([http://fcon_1000.projects.nitrc.org/indi/CoRR/html/hnu_1.html](about:blank)). We included all data points from these datasets that passed our quality and inclusion criteria. Individuals in all used samples were balanced for diagnostic status and nuisance covariates using a propensity score matching technique. The details of the criteria are listed in the method section on pages 18 ff. in the manuscript.

**Replicates**

- You should report how often each experiment was performed
- You should include a definition of biological versus technical replication
- The data obtained should be provided and sufficient information should be provided to indicate the number of independent biological and/or technical replicates
- If you encountered any outliers, you should describe how these were handled
- Criteria for exclusion/inclusion of data should be clearly stated
- High-throughput sequence data should be uploaded before submission, with a private link for reviewers provided (these are available from both GEO and ArrayExpress)

Please outline where this information can be found within the submission (e.g., sections or figure legends), or explain why this information doesn’t apply to your submission:

The stability of subtype metrics was estimated either on the basis of 1000 stratified, random subsamples of the discovery dataset (for subtype maps and discrete assignments), or on longitudinal data taken from the ABIDE 2 and HNU1 datasets (for continuous assignments). Details of these procedures are listed in the section”Stability analysis” on page 21 in the manuscript.

The replicability of association effects between subtypes and ASD diagnosis was tested on an independent validation dataset (ABIDE 2) that was kept separate for this purpose. Details of the replication test are listed in the section “Replicability” on page 24 in the manuscript.

Supplementary analyses investigating the multi-session stability of FC subtypes (Stability analysis, p22), principal components of network FC (Principal component analysis of network FC, p24), and the impact of regressing global functional connectedness (Differences in whole-brain connectivity contribute to FC subtypes, p11) were performed on the same samples as the main analysis. A supplementary analysis investigating the inclusion of cerebellar seeds (Functional connectivity estimation, p20) was performed on a subsample of the data with appropriate cerebellar coverage.

**Statistical reporting**

- Statistical analysis methods should be described and justified
- Raw data should be presented in figures whenever informative to do so (typically when N per group is less than 10)
- For each experiment, you should identify the statistical tests used, exact values of N, definitions of center, methods of multiple test correction, and dispersion and precision measures (e.g., mean, median, SD, SEM, confidence intervals; and, for the major substantive results, a measure of effect size (e.g., Pearson's r, Cohen's d)
- Report exact p-values wherever possible alongside the summary statistics and 95% confidence intervals. These should be reported for all key questions and not only when the p-value is less than 0.05.

Please outline where this information can be found within the submission (e.g., sections or figure legends), or explain why this information doesn’t apply to your submission:

Stability estimates for subtype metrics are reported and interpreted at a descriptive level based on commonly used measures of robustness (ICC, Sørensen–Dice coefficient, Pearson correlation) in the results section on page 3 f. of the manuscript.

Associations between subtypes and ASD diagnosis are tested with general linear models and corrected for multiple comparisons with the Benjamini & Hochberg p-value adjustment. We report adjusted p-values and Cohen’s d effect size measures. Associations on the independent validation dataset are in additon descriptively compared to those obtained on the discovery sample.

(For large datasets, or papers with a very large number of statistical tests, you may upload a single table file with tests, Ns, etc., with reference to sections in the manuscript.)

**Group allocation**

- Indicate how samples were allocated into experimental groups (in the case of clinical studies, please specify allocation to treatment method); if randomization was used, please also state if restricted randomization was applied
- Indicate if masking was used during group allocation, data collection and/or data analysis

Please outline where this information can be found within the submission (e.g., sections or figure legends), or explain why this information doesn’t apply to your submission:

This work does not include an experimental manipulation.

**Additional data files (“source data”)**

- We encourage you to upload relevant additional data files, such as numerical data that are represented as a graph in a figure, or as a summary table
- Where provided, these should be in the most useful format, and they can be uploaded as “Source data” files linked to a main figure or table
- Include model definition files including the full list of parameters used
- Include code used for data analysis (e.g., R, MatLab)
- Avoid stating that data files are “available upon request”

Please indicate the figures or tables for which source data files have been provided:

The unthresholded association tests are provided as source data in the figure supplement to figure 2.

The complete analysis code can be found on github: [https://github.com/surchs/ASD_subtype_code_supplement](about:blank).
